# Toxoplasma infection induces an aged neutrophil population in the CNS that is associated with neuronal protection

**DOI:** 10.1186/s12974-024-03176-7

**Published:** 2024-08-02

**Authors:** Kristina V. Bergersen, Bill Kavvathas, Byron D. Ford, Emma H. Wilson

**Affiliations:** 1 Division of Biomedical Sciences, School of Medicine, University of California, Riverside, Riverside, CA USA; 2 Present Address: College of Medicine, Howard University, Washington, D.C. USA

**Keywords:** Neutrophils, Brain, Chronic infection, *Toxoplasma gondii*, Neuroprotection, Immune response

## Abstract

**Background:**

Infection with the protozoan parasite *Toxoplasma gondii* leads to the formation of lifelong cysts in neurons that can have devastating consequences in the immunocompromised. In the immunocompetent individual, anti-parasitic effector mechanisms and a balanced immune response characterized by pro- and anti-inflammatory cytokine production establishes an asymptomatic infection that rarely leads to neurological symptoms. Several mechanisms are known to play a role in this successful immune response in the brain including T cell production of IFNγ and IL-10 and the involvement of CNS resident cells. This limitation of clinical neuropathology during chronic infection suggests a balance between immune response and neuroprotective mechanisms that collectively prevent clinical manifestations of disease. However, how these two vital mechanisms of protection interact during chronic Toxoplasma infection remains poorly understood.

**Main text:**

This study demonstrates a previously undescribed connection between innate neutrophils found chronically in the brain, termed “chronic brain neutrophils” (CBNeuts), and neuroprotective mechanisms during Toxoplasma infection. Lack of CBNeuts during chronic infection, accomplished via systemic neutrophil depletion, led to enhanced infection and deleterious effects on neuronal regeneration and repair mechanisms in the brain. Phenotypic and transcriptomic analysis of CBNeuts identified them as distinct from peripheral neutrophils and revealed two main subsets of CBNeuts that display heterogeneity towards both classical effector and neuroprotective functions in an age-dependent manner. Further phenotypic profiling defined expression of the neuroprotective molecules NRG-1 andErbB4 by these cells, and the importance of this signaling pathway during chronic infection was demonstrated via NRG-1 treatment studies.

**Conclusions:**

In conclusion, this work identifies CBNeuts as a heterogenous population geared towards both classical immune responses and neuroprotection during chronic Toxoplasma infection and provides the foundation for future mechanistic studies of these cells.

**Supplementary Information:**

The online version contains supplementary material available at 10.1186/s12974-024-03176-7.

## Introduction

Ingestion of the protozoan parasite *Toxoplasma gondii* leads to a lifelong infection characterized by the formation of cysts inside neurons of the brain. Prevalence rates vary, but it is estimated that one third of the world’s population is currently infected with Toxoplasma [1]. While infection is often asymptomatic due to constant pressure of the host immune response, an immunocompromised state can result in Toxoplasmic encephalitis which is fatal if left untreated [2]. The presence of the parasite in the brain leads to recruitment of both innate and adaptive immune cells which are required to control parasite replication while maintaining a balanced tissue environment [3–6].

Innate neutrophils and chemokine-dependent neutrophil recruitment have previously been identified as vital sources of protection against acute Toxoplasma infection [7–10]. Two independent research groups have recently identified a small but defined population of these cells in the brain between 2- and 4-weeks post infection (wpi) [5, 11]. The Dunay group has partially characterized these cells and has also shown that depletion of neutrophils at the start of chronic infection leads to increased parasite burden and inhibited immune cell recruitment to the brain [5]. The presence of neutrophils in the brain during chronic infection suggests a more versatile role of these cells than previously thought. Literature focusing on potential alternative roles for neutrophils in non-Toxoplasma models describes two basic classes of these cells based on their differential expression of markers such as CD15 and CD49d among others [12–14]. More recent works investigating neutrophils in homeostasis, tissue injury, and non-Toxoplasma models of infection have expanded upon this to include a much broader range of previously undescribed neutrophil heterogeneity [15–18]. Therefore, the chronic presence of neutrophils in the brain may point to an important yet undefined role for these innate immune cells in maintaining brain homeostasis during chronic Toxoplasma infection.

Despite cyst-containing neurons and brain inflammation caused by peripheral immune cells like neutrophils, Toxoplasma infection causes few clinical symptoms in the immunocompetent host. However, underlying changes in neurochemistry and significant changes in the health of neurons suggest a constant need to balance inflammation and protect against neuropathology [19, 20]. Recent work in our lab to determine Toxoplasma-induced changes in immunological and neurological transcripts in the brain supports these sub-clinical neurological changes, but also reveals potential activated neuroprotective pathways that remain poorly understood [21]. The concept of “neuroprotection” (prevention of neuronal death and maintenance of neuronal homeostasis during injury/infection) has previously been investigated in models of central nervous system (CNS) injury, such as ischemic stroke and spinal cord injury, and during experimental cerebral malaria (ECM) infection. In these models, neuroprotective functions are activated predominantly via signaling of the endogenous ligand Neuregulin 1 (NRG-1) through its main receptor ErbB4 [22, 23]. Furthermore, resolution of neuropathology in a model of ischemic stroke occurs through the clearance of damage signals, debris, and revascularization by macrophage scavenger receptor 1 (MSR1) [24]. MSR1 transcripts are upregulated during Toxoplasma infection and correlate with increasing chronicity [21]. While the sources of these three molecules and their contribution to neuroprotection have been investigated in other disease models, their cellular sources and roles in protecting against neuropathology during chronic Toxoplasma infection remain unknown.

In this study, we present a previously undescribed connection between “chronic brain neutrophils” (CBNeuts) and neuroprotective mechanisms during chronic Toxoplasma infection. Lack of CBNeuts after chronic infection was well-established led to a decrease in neuronal regeneration suggesting a potential undefined role of these cells in neuroprotection. Phenotypic characterization of CBNeuts identified their expression of neuroprotective molecules including NRG-1, ErbB4, and MSR1 among others. CBNeuts had distinct phenotypic and transcriptomic profiles compared to peripheral neutrophils (PNeuts) including CNS-specific signatures that further distinguish two distinct CBNeut populations. The combined data reveal that CBNeuts are capable of acting simultaneously as immune effector and neuroprotective cells and may play a vital flexible role in maintaining brain homeostasis during chronic Toxoplasma infection.

## Materials and methods

### Animals, parasites, and mouse infections/kinetics studies

*Animals.* All research was conducted in accordance with the Animal Welfare Act, and all efforts were made to minimize suffering. All protocols were approved by the Institutional Animal Care and Use Committee (IACUC) of the University of California, Riverside. Female 6–8 week old WT C57BL/6J mice were obtained from Jackson Laboratories and were maintained in a pathogen-free environment in accordance with IACUC protocols at the University of California Riverside.

*Parasites and Infections.* The ME49 strain of *T. gondii* was maintained in cyst form by continuous passaging in SW and CBA/J mice. Female 6–8 week old WT C57BL/6J mice were infected with 10 ME49 cysts per mouse in 200 µl of sterile 1x PBS solution via intraperitoneal (IP) injection. Naïve controls received 200 µl of sterile 1x PBS solution via IP injection.

Mice were infected as described above and sacrificed at the following acute and chronic time points: 1week post infection (wpi) (an acute time point where little/no cyst formation is seen in the brain), 2wpi (an early chronic time point where cysts are actively forming and immune cells are recruited), 4wpi and 6wpi (2 mid-chronic time points where cysts have been fully formed and a chronic inflammatory state is well-established), and 11wpi (a late chronic time point where increased parasite reactivation can sometimes be seen). At each time point, half brain and whole spleen were harvested for flow cytometry, and the other half brain and one lobe of liver were harvested for immunohistochemistry, Western blot, or parasite burden, respectively. For single cell RNA sequencing experiments, whole brain was used.

### Neutrophil depletion and NRG-1 treatment studies

*Neutrophil Depletion*. After 4 weeks of infection, when chronic infection was well-established, a cohort of infected mice were injected with 500 µg/mouse of Ly6G depleting mouse monoclonal antibody in vehicle (sterile 1x PBS) via 200 µl IP injection every other day for 14 days according to previously published protocols [5]. A cohort of infected control mice received 200 µl/mouse of vehicle (sterile 1x PBS) intraperitoneally every other day for 14 days to control for depletion injections.

*NRG-1 Treatment.* After 2 weeks of infection, when cysts were actively forming in the brain, a cohort of infected mice were injected with 5 µg/kg/day of NRG-1 (R & D Systems) in vehicle (sterile 1x PBS, 0.1% BSA) via 100 µl IP injection every day for 7 days according to previously published protocols [23]. A cohort of infected untreated control mice received 100 µl/mouse of vehicle (sterile 1x PBS, 0.1% BSA) intraperitoneally every day for 7 days to control for treatment injections.

*Western Blot.* Half brains were harvested 24 h after NRG-1 treatment completion and placed in RIPA buffer. Protein was extracted, normalized, and run on a Western blot according to previously published protocols [19]. Blots were imaged separately for GLT-1 and β-Actin as a control and analyzed using ImageJ software.

### Parasite burden and B1 gene analysis

Parasite burden in brain and peripheral liver was quantified as previously described [21, 25]. Briefly, DNA from half brains and liver from naïve, infected control, and infected neutrophil-depleted mice (*n* = 5/group) was extracted and purified using a High Pure PCR Template Prep Kit (Roche). DNA concentration of each sample was determined via NanoDrop, and all DNA was normalized to 12.5 ng/µl before amplification. Parasite burden was measured by amplifying the B1 gene of *T. gondii* by RT-PCR.

### Cell processing for flow cytometry and cell sorting

Naïve and infected female C57BL/6J mice were sacrificed and perfused intracardially with 20 mL of sterile 1x PBS. Blood, spleens and half brains were harvested, and splenocytes and brain mononuclear cells (BMNCs) were processed according to previously published protocols [19, 25]. For neutrophil localization studies, whole brain was harvested and dissected into 3 broad regions (cortex, mid-brain region, and cerebellum) using brain morphology to define regions for each mouse. BMNCs were then processed as above.

### Flow cytometry

Processed cells were diluted with FACS buffer (4 g BSA, 50 mg EDTA, 1 L 1xPBS) to 1.0 × 10^6^ cells/ml (or all cells from respective brain regions) and transferred to FACS tubes for staining. Cells were incubated with 1:10 FC Block (BD) for 10 min on ice followed by fluorophore-conjugated or primary unconjugated antibodies for surface staining for 30 min protected from light. Cells were washed with FACS buffer solution and incubated in fluorophore-conjugated secondary antibodies as needed for 30 min protected from light. Fluorophore-conjugated antibodies used during surface staining are as follows: CD45 PE (Invitrogen), CD11b APCCy7 (Invitrogen), Ly6G PerCPcy5.5 (Clone 1A8, BD), CD62-L APCCy7 (BD), CD3 FITC (BD), CD4 APC (Invitrogen), CD4 APCCy7 (Invitrogen), CD8 PECy7 (Invitrogen), CD11b PerCPCy5.5 (eBioscience), CD11b APC (eBioscience), Ly6G BV510 Clone 1A8 (eBioscience), CXCR4 PECy5.5 (ThermoFisher), and MMP9 AF647 (SantaCruz Biotech). Primary unconjugated antibodies and their corresponding fluorophore-conjugated secondary antibodies are as follows: NRG-1 primary antibody (Invitrogen) with Alexafluor 647-conjugated secondary antibody (ThermoFisher); NRG-1 primary (Santa Cruz Biotech) with Alexafluor 568-conjugated secondary (ThermoFisher); MSR1 primary (Invitrogen) with Alexafluor 488-conjugated or Q Dot 655-conjugated secondary (ThermoFisher); VEGF primary (NovusBio) with Alexafluor 680-conjugated secondary (ThermoFisher); and biotinylated CD15 primary (Invitrogen) with PECy7-conjugated Streptavidin (eBioscience). Following surface staining, cells were washed, fixed in 4% PFA, and resuspended in FACS buffer. For intracellular staining, cells were spun at 1500 rpm in 0.3% Saponin for 10 min to permeabilize cells and then incubated with FITC-conjugated ErbB4 (Santa Cruz Biotech) in Saponin to maintain permeabilization. After incubation, cells were washed and resuspended in FACS buffer. Samples were acquired using either a BD FACS Canto II flow cytometer or NovoCyte Quanteon machine, NovoSampler Q, and NovoExpress Software at the UC Riverside core facility. Analysis was conducted using FlowJo software. For specific panels and gating strategy used, see Supplementary Tables 1 and Supplementary Fig. 1.

### Cell sorting and single cell RNA sequencing

*Cell Sorting.* Neutrophils were sorted from brain and spleen based on CD11b/Ly6G positivity. BMNCs and splenocytes were harvested as above at 4wpi. To obtain a large enough number of neutrophils for sequencing, 3 naïve and 3 infected mice were pooled for naïve and infected neutrophil splenocyte samples, and 7 infected mice were pooled for neutrophil BMNC sample. 3 separate infected mice were pooled for an unsorted BMNC control sample. Cells were incubated in fluorescent-conjugated antibodies for CD11b APCCy7 (Invitrogen) and Ly6G PE or PerCPCy5.5 (Clone 1A8; Invitrogen), rinsed, and resuspended at 1.0 × 10^7^ concentration in FACS buffer with 10% FBS to minimize cell death during sorting. Neutrophils were sorted from pooled samples based on gating strategy of Ly6G + CD11b + cells using a MoFlo Astrios EQ Cell Sorter at 3–4% pressure to maximize cell survival. Sorted cells were re-counted to determine viability, and a minimum of 1.0 × 10^4^ sorted cells were used for single cell sequencing.

*10x Sequencing and Analysis.* Sorted Ly6G + CD11b + cells and unsorted BMNC control sample were processed for single cell RNA sequencing according to 10x sample prep protocols for Steps 1–3 (Manual: CG000204 RevD); instructions were followed exactly. Time spent before loading sorted cells onto 10x Chromium controller for Step 1 was < 1 h. Processed samples were analyzed after Steps 2 and 3 via Bioanalyzer by the UCR Genomics Core facility for viability and concentration before proceeding to next steps. Upon completion of the 10x protocol, samples were sent to the UC San Diego IGM Genomics Center for sequencing (500 million reads/sample).

### Immunohistochemistry and Immunofluorescence

A minimum of 4 biological replicates were used for each group of mice (naïve, infected control, and infected neutrophil-depleted OR infected NRG-1-treated). Following perfusion, half brains were harvested and post-fixed in 4% PFA for at least 24 h followed by 30% sucrose for 48 h. Brains were frozen at -80 °C in optimal cutting temperature compound (OCT) and sectioned sagittally at 10 μm thickness using a cryostat and charged slides.

*H&E Staining*. H&E staining protocol was as follows: fixation of slides in 95% ethanol, incubation in hematoxylin followed by eosin stain for 30 s each (with fixation step in between), fixation in 95% and 100% ethanol, and final fixation using Citrisolv. Slides were sealed with coverslips using Cytoseal (ThermoFisher) and dried overnight. Imaging was performed using ImageJ software.

*Cyst Quantification.* For quantification of cysts from H&E-stained brain sections, quantification was blinded. Total numbers of cysts were quantified from each biological sample (*n* = 5 per group, 4 brain slices per sample). Total counts per biological sample were calculated by adding numbers of cysts counted in each brain slice to account for biological variability in each group.

*Immunofluorescence Staining*. Slides were fixed and permeabilized in 75% acetone/25% ethanol for 10 min at room temperature. Slides were blocked with 5% Donkey serum for 30 min at room temperature and incubated with primary antibodies at room temperature for 1 h protected from light. Primary antibodies and dilutions used are listed: Goat *T. gondii* (1:300, Abcam); Rabbit SLPI (1:200, Novus Biologicals); and Guinea pig GLT-1 (1:200, Abcam). After primary incubation, slides were rinsed three times with 1x PBS for 5 min each wash and incubated for 1 h protected from light at room temperature with the following secondary antibodies (1:1000 dilution, Thermofisher): Donkey anti-Rabbit Alexafluor 488, Donkey anti-Goat Alexafluor 568, and Chicken anti-Guinea pig Alexafluor 488. For specific panels used, see Supplementary Table 2. Following secondary incubation, slides were washed 3x in 1x PBS for 5 min each wash. Coverslips were mounted on slides using VectaShield Hardset Mounting Medium with DAPI (Vector Labs), and slides were dried overnight in the dark at room temperature. Imaging was performed using a Leica inverted DMI6000 B microscope and Las-X software.

*SLPI Quantification*. For quantification of SLPI + cells, slides were blinded, and a minimum of 10 positively stained cells were counted (from a minimum of 5 regions of interest (ROIs) per section x 4 sections/ biological sample = minimum of 20 ROIs per biological sample) from the cortex region of the brain (where the majority of cyst formation occurs).

SLPI-positive staining of neuronal axons was quantified. Other cell types such as astrocytes can express SLPI, but NeuN staining could not be used for colocalization with SLPI expression on neuronal axons due to restriction of NeuN to the neuronal nucleus. Therefore, only SLPI + neuronal axons were identified/quantified based on accurate cortical neuron-axon morphology and different morphology/staining from SLPI + astrocytes seen in previous studies [26, 27].

### Statistical analyses

All experiments were repeated a minimum of 3 times to confirm accuracy and consistency of results, and all experiments were conducted with a minimum of *n* = 3 to account for biological variability. Statistical significance for all experiments was determined by either 2- tailed unpaired Student’s t-test or One-Way ANOVA with multiple comparisons, and a p-value < 0.05 was considered statistically significant. The type of statistical test run for each experimental result is indicated in the corresponding figure legends.

*scRNAseq Analysis*. Sequencing files were analyzed using the following softwares: FileZilla, HPCC Cluster, Cell Ranger (version 5.0), and Loupe Browser (version 5.0). FastQC and MultiQC were used to evaluate the quality of sequencing reads. Sequencing reads were aligned (fastq) to the mm10 mouse reference genome for analysis, and the expression of transcripts was quantified in each cell. Low quality cells were excluded from analysis using the Normalize function during analysis. Sequencing saturation was confirmed for each sample. After quality control checks, the following cells and average gene reads per cell were obtained: Brain Neutrophils = 3,242 cells, 736 reads/cell; Infected Spleen Neutrophils = 6,804 cells, 782 reads/cell; Naïve Spleen Neutrophils = 4,813 cells, 702 reads/cell; BMNCs = 2,280 cells, 1,014 reads/cell. For differentially expressed genes (DEGs), genes with *p* < 0.05 after comparison to control values were considered significantly enriched, and all genes discussed in this study were identified as significantly altered. Selected genes for heatmap analysis were confirmed as significantly altered (*p* < 0.05). The top 50 most significantly enriched genes in each identified neutrophil subset were used for Gene Ontology (GO) analyses, and the “Biological Processes” option was selected for the *Mus musculus* host.

## Results

### Neutrophils persist in the brain throughout chronic infection

**Fig. 1:**
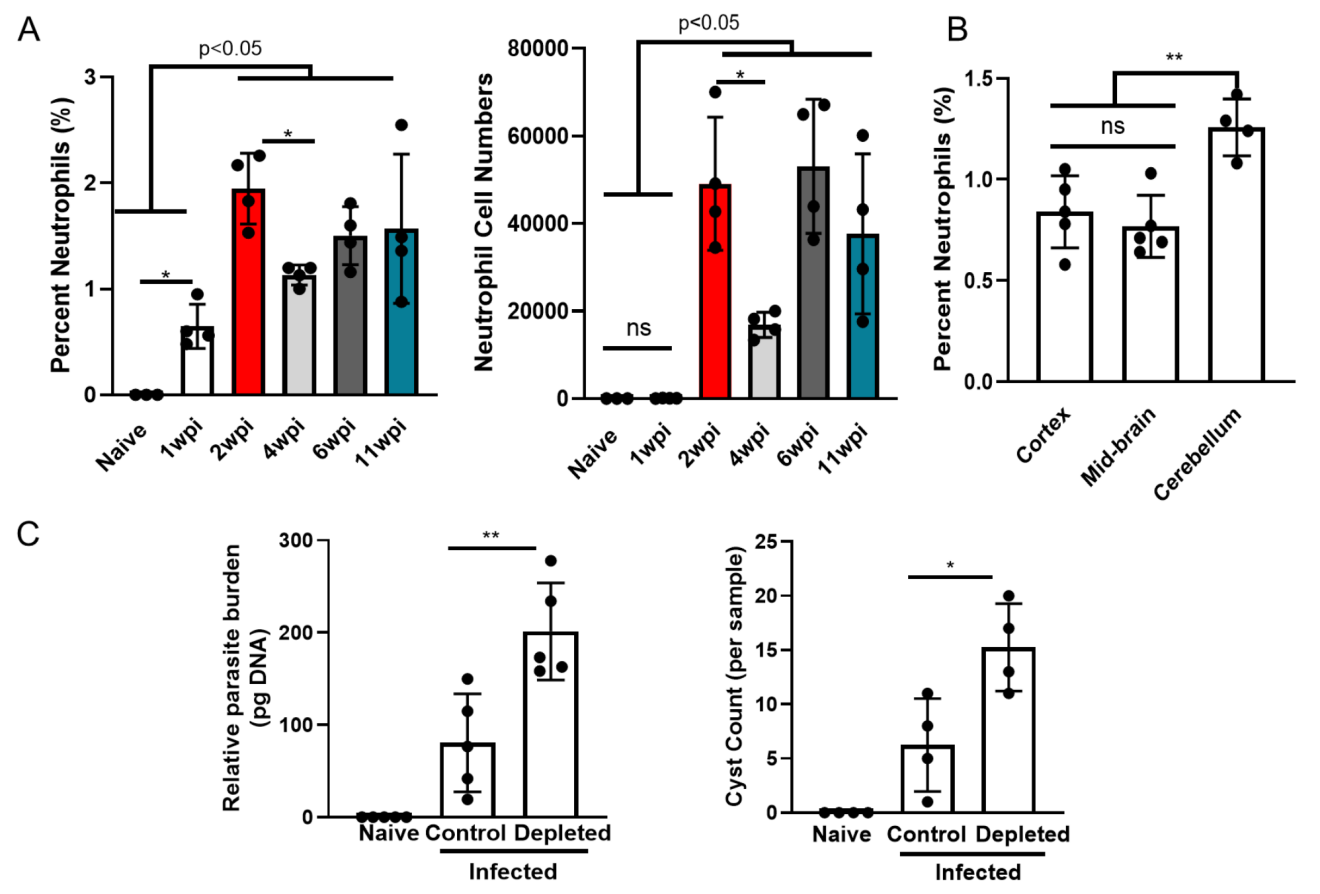
Neutrophil persistence in the brain during chronic infection is vital for control of parasite replication. C57BL/6J mice were infected intraperitoneally with 10 *T. gondii* cysts (*n* = 4 per time point) or injected with PBS as a control (*n* = 3) and analyzed for immune cells in the brain at both acute (1wpi) and chronic (2, 4, 6, and 11wpi) time points via flow cytometry. For location studies, mice (*n* = 5) were sacrificed at 4wpi, brains were dissected into 3 distinct regions, and BMNCs were analyzed via flow cytometry to determine neutrophil location in the brain. For neutrophil depletion, a cohort of infected mice received neutralizing Ly6G mAb treatment at 4wpi for 2 weeks to deplete neutrophils. (**A**) Frequencies (left) and numbers (right) of CD11b + Ly6G + neutrophils in brain at each time point. (**B**) Quantification of neutrophil percentages in each defined brain region. (**C**) Quantified parasite burden from whole brain DNA via RT-PCR using *T. gondii* B1 gene (left) and cyst burden quantified via direct counting from H&E-stained slides (right). * = *P* < 0.05, ** = *P* < 0.01, “p-val < 0.05” indicates varying degrees of significance between indicated time points; significance determined via One-way ANOVA (**A**-**B**) or unpaired student t-test (**C**), and error bars indicate SD

As previously demonstrated [5], a small, well-defined population of neutrophils (Ly6G^hi^, CD11b^+^) was seen in the brain during chronic infection (Supplemental Fig. 1) that were fully differentiated and mature based on their expression of CD11b, Ly6G, MHC I, and the lack of the proliferative marker Ki67 (Supp. Figure 1B). To determine the size and duration of this population over the course of infection, neutrophil percentages and absolute cell numbers were quantified from the brain tissue at 1, 2, 4, 6, and 11wpi. Neutrophils were found within the brain parenchyma as early as 1wpi with peak infiltration seen at 2wpi (Fig. 1A). These are time points when the cell lytic tachyzoites are invading the brain prior to cyst formation [21]. A significant contraction of neutrophil numbers was observed at 4wpi, however, by late chronic stage, the number of neutrophils increased back to levels comparable with early chronic (2wpi) infiltration (Fig. 1A). Previous studies during chronic Toxoplasma infection have found specific localization of infiltrating inflammatory monocytes to the olfactory tubercle [11], and non-infection models have previously demonstrated the presence of neutrophils in the olfactory bulb [28]. To determine the potential localization of neutrophils to a particular part of the CNS, brains were perfused and the cerebellum, frontal cortex and mid-brain were dissected, anatomical areas that are easily identified and isolated. The percentage of neutrophils was elevated in the cerebellum although absolute neutrophil numbers showed little region specificity implying that neutrophils may access the cerebellum more easily than other immune cells (Fig. 1B and data not shown). These results indicate a broad disbursement of neutrophils in the brain during infection instead of localization to specific brain regions. Following previous work by the Dunay group [5], neutrophils were depleted over the course of 2 weeks starting at 4wpi using a neutralizing Ly6G mAb (Fig. 1C; Supplemental Fig. 2) and parasite burden was measured. In the absence of neutrophils, Toxoplasma *B1* gene analysis and cyst burden was significantly elevated (Fig. 1C). These results demonstrate an essential, persistent, and broadly disbursed chronic neutrophil population in the brain, which we termed “CBNeuts.”

### CBNeuts are defined by distinct transcriptional and protein signatures that confirm their tissue specificity

**Fig. 2:**
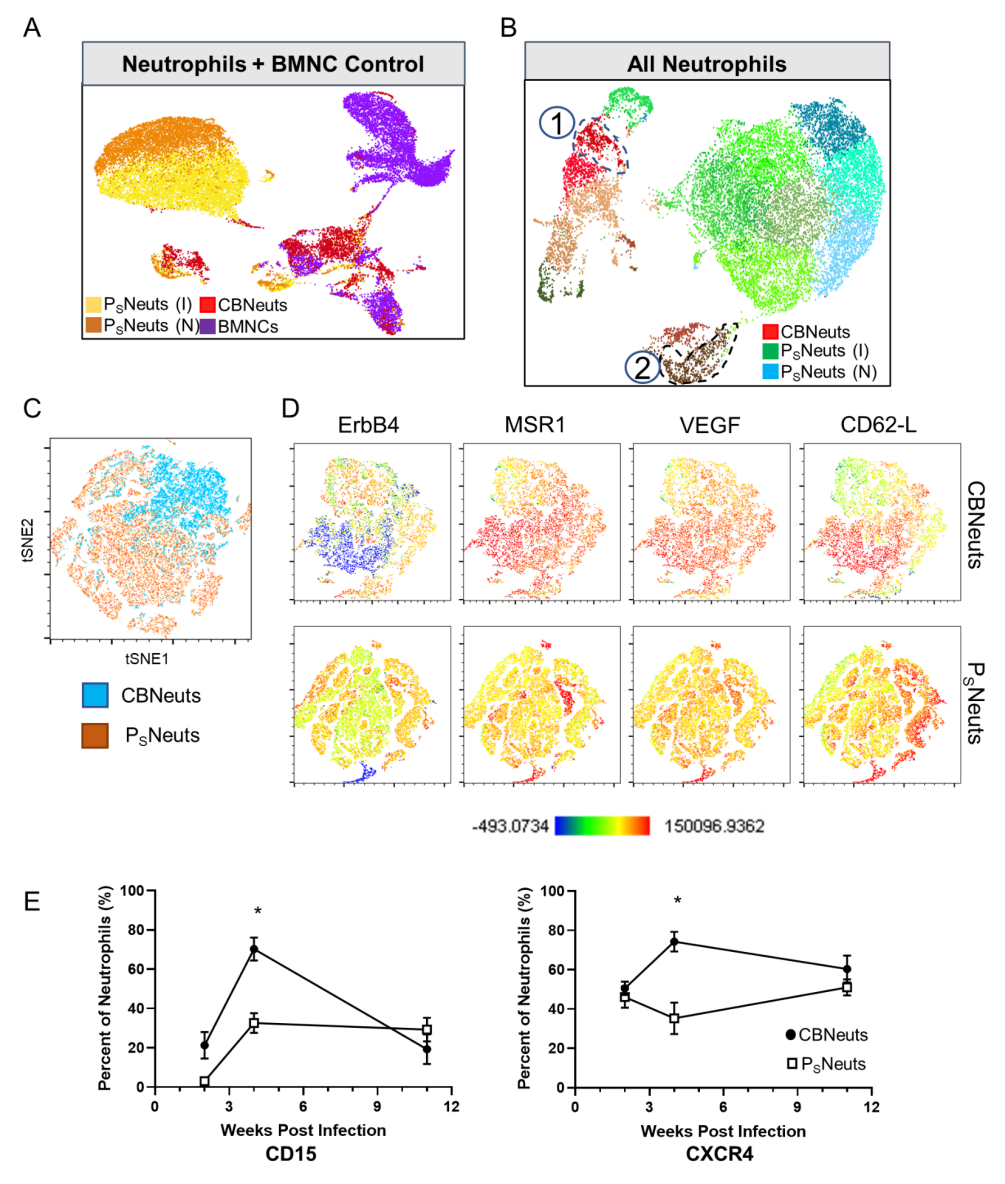
Transcriptomic and protein analysis of CBNeuts reveals a distinct tissue-specific profile. For transcriptomic profiling, CBNeuts (from *n* = 7 brains) and P_S_Neuts (from *n* = 3 spleens) were sorted from infected mice at 4wpi via flow cytometry and prepped for scRNAseq along with chronically infected BMNCs (*n* = 3 mice) and naïve spleen neutrophil (*n* = 3) controls. Phenotypic profiles from CBNeuts and P_S_Neuts were also evaluated via flow cytometry at 4wpi. For both, neutrophils were identified based on expression of CD11b and Ly6G. (**A**) UMAP plot of all aggregated scRNAseq samples: P_S_Neuts from infected mouse (I, yellow), P_S_Neuts from naïve mouse (N, orange), CBNeuts (red), and BMNCs (purple). (**B**) UMAP plot of aggregated sorted CBNeuts, P_S_Neuts from infected mouse spleen (I), and P_S_Neuts from naïve spleen (N). (**C**) tSNE plot of concatenated CBNeuts and P_S_Neuts at 4wpi. (**D**) tSNE plots of selected alternative molecules from concatenated CBNeuts (top) and P_S_Neuts (bottom). tSNE plot scale shows populations with low expression (blue) to high expression (red) of molecules. (**E**) Flow cytometry quantification of CD15 (left) and CXCR4 (right) expression by CBNeuts and P_S_Neuts at multiple chronic infection time points (2, 4, and 11wpi). * = *p* < 0.05; significance determined via unpaired student t-test, and error bars indicate SD

The landscape and immune requirements of neutrophils during chronic infection are potentially very different from the environment of acute peripheral inflammation normally associated with neutrophil function [29]. This, in addition to our increasing understanding of trained immunity and the role of neutrophils in the brain [30–34] provoked us to investigate the transcriptional and phenotypic profiles of CBNeuts to determine if neutrophils that have entered the brain have a unique signature. To do so, single cell RNA sequencing (scRNAseq) and flow cytometry were performed on sorted CBNeuts and peripheral neutrophils (P_S_Neuts) from infected and uninfected spleens to assess tissue-specific gene and protein signatures. Cells were sorted from pooled brains and spleens based on positive CD11b and Ly6G expression. Resulting 4-week-infected samples were aggregated following scRNAseq, and transcriptomic profiles of CBNeuts vs. P_S_Neuts were analyzed (Fig. 2A-B). *Ly6G* gene enrichment was evaluated in all sequenced samples (Supplemental Fig. 3A) to confirm neutrophil specificity. When comparing all aggregated samples, CBNeuts clustered separately from splenic neutrophils and control unsorted BMNC cells (Fig. 2A). When all sorted neutrophils were aggregated and analyzed separately, a nearly complete separation of CBNeuts (reds) was observed compared to P_S_Neuts from infected (greens) and naïve (blues) mice (Fig. 2B).

Based on the distinct separation of neutrophils from the brain compared to the spleen seen in our scRNAseq data, CBNeuts and P_S_Neuts were further analyzed at 4wpi for the expression of proteins known to play a role in neutrophil function (Fig. 2C-E). Previously identified proteins including MMP9, VEGF, CD62-L, CD15 and CXCR4 are found in peripheral neutrophils with a more alternative/protective function, including tissue repair and angiogenesis [5, 15]. To determine the expression of these molecules previously associated with protective neutrophil functions and general neuroprotection (NRG-1, ErbB4, and MSR-1) by our CBNeut population, BMNCs and peripheral splenocytes were incubated with antibodies to Ly6G, CD11b, and the proteins mentioned above, and multi-parameter flow cytometry was conducted. When CBNeuts were compared to P_S_Neuts as a whole, tSNE plots revealed mostly non-overlapping populations (Fig. 2C). Striking visual differences between CBNeuts and P_S_Neuts were observed in the expression of ErbB4, MSR1, VEGF, and CD62-L (Fig. 2D), with CBNeuts uniformly expressing MSR1 and VEGF compared to P_S_Neuts. tSNE clustering defined CBNeut subsets based on noticeable differential expression of ErbB4 and CD62-L. While populations of these defined subsets were also seen in P_S_Neuts, they made up a much smaller proportion of the total population compared to CBNeuts. NRG-1 expression did not further define CBNeut subsets but was expressed uniformly (data not shown). Analysis of neutrophils that were migrating in the blood served as another peripheral population and demonstrated similar results to those seen in P_S_Neuts (data not shown).

Neutrophils can also be distinguished by age and changes in migratory capability as reviewed by Peiseler and Kubes [15]. Thus, it was hypothesized that differences in migration capability and age would be apparent in CBNeuts compared to P_S_Neuts as chronic infection progressed. To address this, the expression of CD15 (indicative of the ability to migrate [14]) and CXCR4 (indicative of aged neutrophils [35]) was monitored over the course of chronic infection (Fig. 2E). The percent of CD15 + CBNeuts significantly increased from 2wpi (20%) to 4wpi (> 60%) and dropped back to baseline by 11wpi (20%) (Fig. 2E, left). In contrast, the proportion of CD15 + P_S_Neuts increased less from 2-4wpi and remained constant (~ 30% of whole population) from 4wpi on. CXCR4 expression followed a similar trend in CBNeuts, significantly increasing from 2- to 4wpi, however remained consistently high through 11wpi (Fig. 2E, right). P_S_Neuts had the inverse relationship showing a decrease in the proportion of CXCR4 expression between 2-4wpi (Fig. 2E). These results confirm the hypothesized differences in migration capability and age between CBNeuts and P_S_Neuts over time.

Collectively, these results identify CBNeuts as transcriptionally and phenotypically distinct from P_S_Neuts based on the expression of protection/repair-associated proteins, migratory capability, and age.

### CBNeuts are composed of two distinctly characterized subsets

**Fig. 3:**
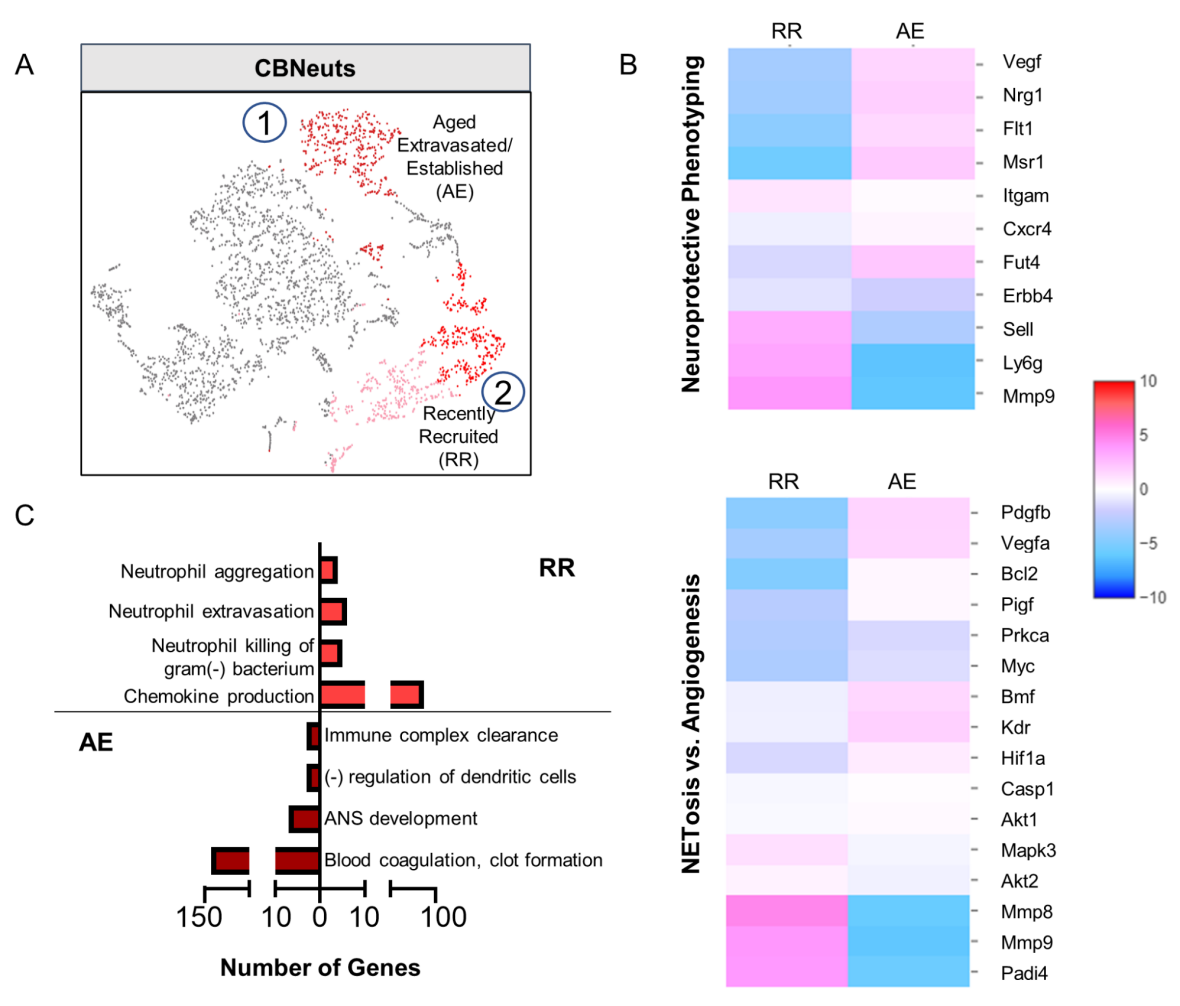
CBNeuts display age-dependent heterogeneity at the transcriptomic level. CBNeuts (from *n* = 7 brains) were sorted from infected mice at 4wpi via flow cytometry and prepped for scRNAseq. A) UMAP plot of CBNeuts (red = neutrophil populations) identified as: (1) “Aged Extravasated” (AE) (*Ly6G*^int^, *Fut4*+, *CXCR4*+) or (2) “Recently Recruited” (RR) (*Ly6G*^hi^, *Fut4*^int^, *CXCR4*-) subsets. Subsets also identified in aggregated dataset (Fig. 2B). Pink subset identified as red blood cells. **B**-**C**) Heat maps of (**B**) neuroprotective genes (top panel) and genes relating to classical (NETosis) vs. alternative (angiogenesis) functions (bottom panel). (**C**) GO enrichment terms from top 50 most significantly upregulated genes in AE and RR CBNeuts

Having already identified distinct phenotypic characteristics of CBNeuts, it was also clear from our global scRNAseq data that these neutrophils were not homogeneous. Indeed, from our scRNAseq analysis of sorted CBNeuts, two distinct clusters (marked as “1” and “2”) were confidently identified as neutrophils based on their enrichment of *Ly6G* and *Itgam* (encodes CD11b) (Fig. 3A). Importantly, these clusters could also be identified in our original aggregated dataset (Fig. 2B). Remaining cell types not identified as “neutrophils” were noted for the expression of lineage-specific markers (T cells, macrophages/microglia, inflammatory monocytes, neurons, astrocytes, and oligodendrocytes; Supplemental Fig. 3B) and were excluded from further analysis. An additional potential neutrophil population (pink) was later identified as red blood cells (Supp. Figure 3B) and was also excluded from further analysis. The two remaining CBNeut clusters were further classified based on enrichment of *Fut4* (encodes for CD15) and *Cxcr4* [15]. Cluster 1 (Fig. 3A, dark red) was enriched for *Fut4* and *Cxcr4* and was termed “Aged Extravasated” (AE). Cluster 2 (Fig. 3A, light red) showed enrichment for *Fut4* but not *Cxcr4* and was termed “Recently Recruited” (RR).

To determine the genes that define these two subsets of CBNeuts, analysis of differential gene expression was conducted using RR CBNeuts as the control group. “Neuroprotective Phenotyping” heatmap analysis demonstrated enrichment for *Vegfa*, *Nrg1*, *Flt1* (encodes for VEGF receptor), and *Msr1* in AE cells, while RR cells showed enrichment for *Sell* (encodes for CD62-L) and *Mmp9* and had increased *ErbB4* compared to the AE subset (Fig. 3B). In contrast, analysis of gene enrichment corresponding to classical NETosis vs. alternative angiogenesis functions found enrichment of genes relating to both processes in each subtype (Fig. 3B). AE cells demonstrated enrichment for *Bmf* (NETosis gene) and the angiogenic genes *Pdgfb*, *Vegfa*, *Kdr*, and *Hif1a*; and RR cells were enriched for the NETosis-associated genes *Mapk3* and *Padi4* and the angiogenic genes *Mmp8* and *Mmp9*. Specific indicators of apoptosis were also examined including *Bcl2*, *Casp1*, *Akt1*, and *Akt2* and showed no significant enrichment in either subset indicating no cell death in either subset.

GO analysis of the top 50 most enriched genes in each CBNeut subset revealed differential upregulation of GO terms (Fig. 3C). AE cells were most enriched for GO terms relating to negative immune regulation and neural development, while RR cells were enriched for terms associated with classical antimicrobial and inflammatory neutrophil functions. Taken together, these results demonstrate that CBNeuts are composed of two major age-dependent subsets that can be distinguished by their expression of neuroprotective genes and transcriptionally-predicted functions.

### CBNeuts are capable of limiting infection-induced neuropathology, which may be partially dependent on NRG1 signaling

**Fig. 4:**
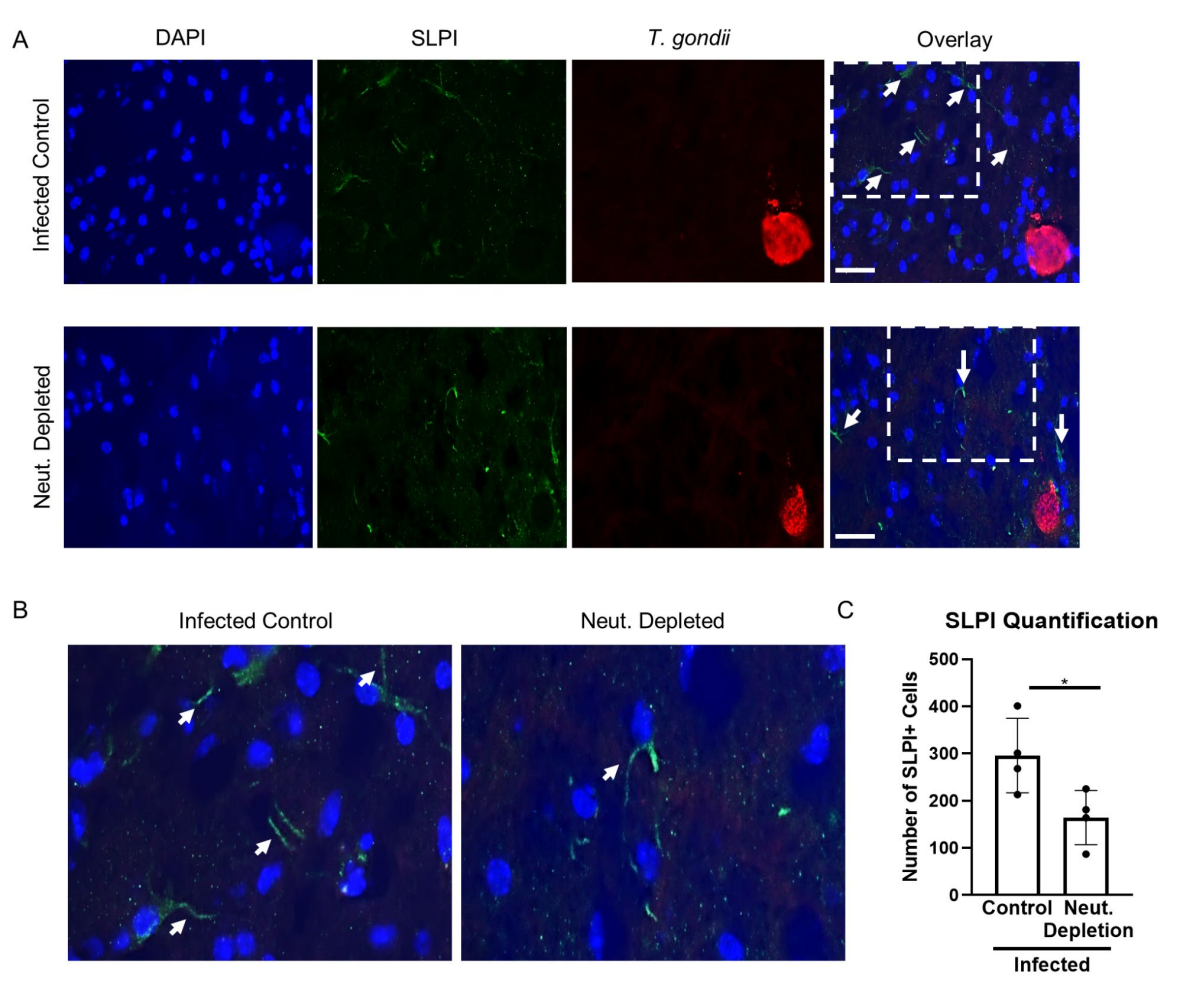
Neuronal regeneration attempts during chronic infection are inhibited in the absence of neutrophils. C57BL/6J mice were infected intraperitoneally with 10 *T. gondii* cysts or injected with PBS as a control (*n* = 5 per group), and a cohort of infected mice received neutralizing Ly6G mAb treatment at 4wpi for 2 weeks to deplete neutrophils. (**A**) Immunofluorescence images of infected control and neutrophil-depleted brains, 40x images. Blue = DAPI, Green = SLPI, Red = *T. gondii*, Scale bar = 25 μm. White arrows indicate SLPI + cells. (**B**) Magnified images of selected regions (dashed white boxes) in (**A**) to show SLPI stained neuronal axons. (**C**) Blinded quantification of SPLI + cells. *=*P* < 0.05; significance determined via unpaired student t-test, and error bars indicate SD

Neutrophils in the CNS have been previously seen encouraging neuronal regeneration [36, 37]. This occurs partially via the production of secretory leukocyte protease inhibitor (SLPI) [26, 38]. To determine whether neutrophils perform similar functions during Toxoplasma infection, we investigated SLPI expression in the brain following depletion of neutrophils via immunofluorescence of mouse brain tissue (Fig. 4). SLPI expression could be seen in the infected brain with characteristic morphology of neuronal bodies and axons which were classified as “SLPI + cells”. SLPI + neuronal cell bodies and SLPI + axons (shown by white arrows) were identified in both infected control and neutrophil-depleted brains (Fig. 4A-B). When this data was blindly quantified, mice lacking neutrophils had significantly fewer SLPI + cells when compared to infected control mice independent of areas of parasite replication (Fig. 4A and C), supporting a neuroprotective phenotype for neutrophils in the CNS.

The persistence of CBNeuts and the results from our neutrophil depletion studies suggested a previously unknown role of neutrophils in maintaining brain homeostasis during chronic infection via neuroprotective mechanisms. Previous work in our lab has also demonstrated the activation of potential neuroprotective and reparative pathways in the brain during chronic Toxoplasma infection [21], but the cellular sources of molecules involved in these pathways remain unknown.

**Fig. 5:**
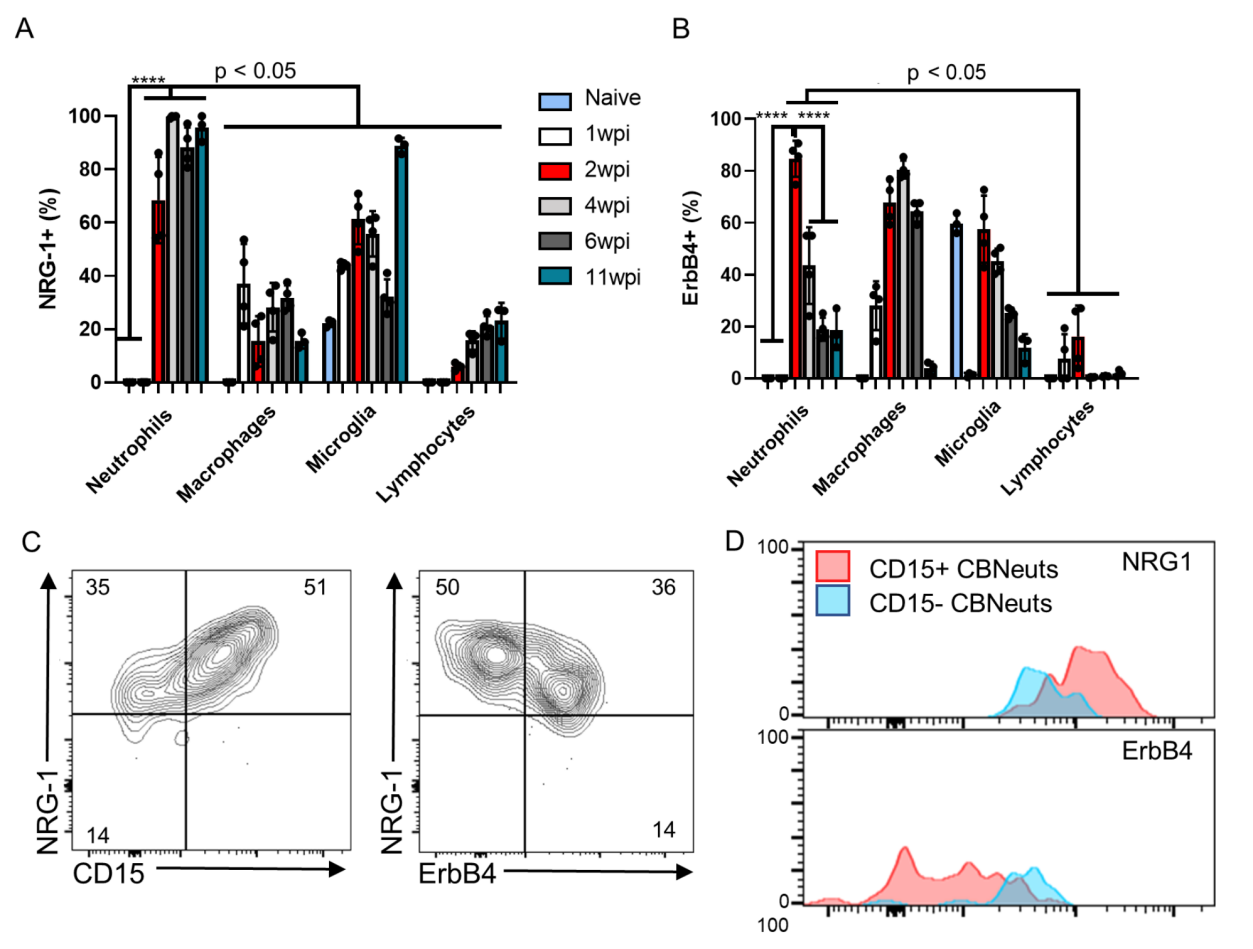
CBNeuts express neuroprotective molecules NRG-1 and ErbB4. C57BL/6J mice were infected intraperitoneally with 10 *T. gondii* cysts (*n* = 4 per time point) or injected with PBS as a control (*n* = 3) and analyzed for immune cells in the brain and expression of neuroprotective molecules at both acute (1wpi) and chronic (2, 4, 6, and 11wpi) time points via flow cytometry. For NRG-1 treatment, a cohort of 2 week-infected mice were treated with 5 µg/kg/day of NRG-1 daily for 7 days. (**A**-**B**) Time course quantification of expression frequencies (shown by percent of positive cells) of NRG1 (**A**)and ErbB4 (**B**) by brain mononuclear cells (BMNCs). (**C**) Representative flow plots of NRG-1 and ErbB4 expression by CBNeuts at 4wpi. Numbers on flow plots represent the average percentage of expression (*n* = 4). (**D**) Expression overlap of neuroprotective molecules distinguished by CD15+ (pink) and CD15- (blue) CBNeuts. For graphs, ****=*P* < 0.0001 (remaining significance indicated as *p* < 0.05); significance determined via One-way ANOVA. For all graphs, error bars indicate SD

One of the most striking differentiating molecules in the Aged Extravasated CBNeut population was the high enrichment of *Nrg1* (Fig. 3B) which encodes for the NRG-1 protein that is responsible for a large majority of neuroprotective functions in the brain [22, 23, 39, 40]. Neutrophils have not been previously documented as being sources of NRG-1 therefore, to confirm this observation, we performed flow cytometry on BMNCs over the course of infection and examined NRG-1 expression (Fig. 5). Analysis revealed that CBNeuts were consistent, significant, and almost uniform cellular sources of NRG-1 (> 60%) at all chronic infection time points (Fig. 5A) which corresponds with the results seen in our initial tSNE analysis. As previously seen (Fig. 2D), a percentage of CBNeuts also expressed the main NRG-1 receptor ErbB4 (20–60%), and the kinetics revealed a striking high percentage of neutrophils expressing ErbB4 at 2wpi that significantly dropped as infection progressed (Fig. 5B). Innate macrophages and CNS-resident microglia also expressed these molecules while adaptive T cells did not express ErbB4 at any time point; however, a subpopulation of T cells (10–20%) did express NRG-1 which increased over the course of infection (Fig. 5A).

Based on the consistent expression of these two neuroprotective molecules by the majority of CBNeuts, we wanted to elucidate the relationship between neuroprotective molecule expression and the classical versus alternative phenotypes seen in our earlier transcriptional and phenotypic profiling. To do this, we further characterized these cells by looking at NRG-1, ErbB4, and CD15 protein expression together. At 4wpi, more than 80% of all CBNeuts were NRG-1 + and were composed of two main subtypes based on their expression of CD15 (Fig. 5C, left and D, top). This integrin aids in the migration of neutrophils into tissues and has previously been used to identify broad “classical” (CD15+) and “alternative” (CD15-) categories [14] with classical neutrophils primed as effector cells for pathogen killing and alternative neutrophils associated with healing, tissue remodeling, and vascular repair. Surprisingly, 51% of NRG-1 + cells expressed CD15 indicating their ability to migrate, with high expression of NRG-1 correlating with expression of CD15. These two subtypes were further distinguished by their differential expression of ErbB4. Alternative neutrophils that did not express CD15 were more likely to express ErbB4 (indicated by lower NRG-1 expression) and therefore be responsive to NRG-1 (Fig. 5C, right and D, bottom). Collectively, these results identify CBNeuts as consistent cellular sources of NRG-1 that are composed of 2 main subtypes based on their differential expression of CD15 and ErbB4. The novel expression of NRG-1 and ErbB4 by CBNeuts also suggested an important yet undescribed role for NRG-1/ErbB4 signaling in limiting Toxoplasma-induced neuropathology.

**Fig. 6:**
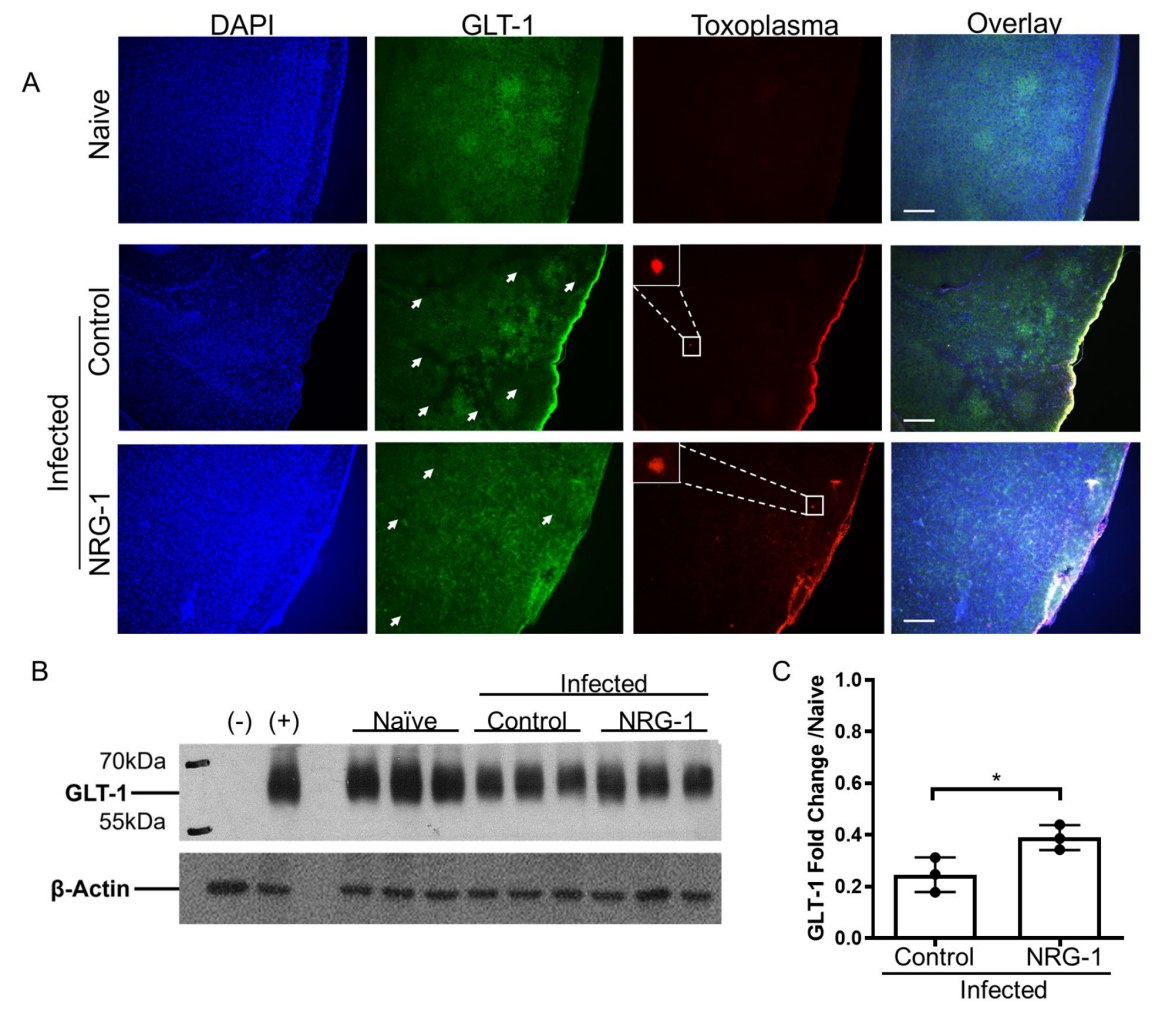
NRG-1 treatment rescues infection-induced GLT-1 levels associated with neurotoxicity. C57BL/6J mice were infected intraperitoneally with 10 *T. gondii* cysts (*n* = 4 per time point) or injected with PBS as a control (*n* = 3). For NRG-1 treatment, a cohort of 2 week-infected mice were treated with 5 µg/kg/day of NRG-1 daily for 7 days. (**A**) Immunofluorescence images of naïve, infected control, and infected NRG-1-treated mouse brain slices. DAPI (blue), GLT-1 (green), and Toxoplasma (red); white arrows indicate areas of decreased GLT-1 expression. Images taken at 10x magnification, Scale bar = 100 μm. (**B**) Western blot of GLT-1 from brain homogenate of naïve, infected control, and infected NRG-1-treated mice. Half brains were harvested and protein was extracted, normalized, and run on a Western blot. (**C**) Quantification of Western blot results using GLT-1:β-Actin ratios. * = *P* < 0.05; significance determined via unpaired student’s t-test., For all graphs, error bars indicate SD

A pathology previously documented in the brain during Toxoplasma infection is the downregulation of GLT-1, a critical glutamate transporter expressed by astrocytes [19]. A build-up of glutamate caused by GLT-1 downregulation leads to neurotoxicity and cell death. To determine the importance of the NRG-1/ErbB4 signaling pathway during chronic Toxoplasma infection, we treated chronically infected mice with exogenous NRG-1 and looked for the presence of GLT-1 downregulation in the infected brain [19] via immunofluorescence and western blot. In naïve mice, GLT-1 was expressed relatively uniformly throughout the brain, but as previously published, GLT-1 loss was seen in “patches” across the tissue during chronic infection (Fig. 6A, white arrows). While it did not return GLT-1 expression to baseline uninfected levels, administration of exogenous NRG-1 treatment significantly rescued infection-induced downregulation of GLT-1, with fewer GLT-1 negative patches and more uniform expression seen in the tissue following treatment (Fig. 6A). This was also quantified at the protein level via western blot, and NRG-1 treatment significantly enhanced the amount of GLT-1 in the brain compared to infected controls (Fig. 6B, C). Collectively, these results highlight the importance of NRG-1/ErbB4 signaling during chronic Toxoplasma infection and offer a potential pathway of neuroprotection employed by CBNeuts and other NRG-1-expressing cells.

## Discussion

The maintenance of a homeostatic brain environment is critically important across a wide range of neurological and immunological diseases. However, the mechanisms behind this are poorly understood during infection where the need to control the pathogen through the immune response must be balanced with maintenance of neuronal function. Under most circumstances, Toxoplasma is an infection that does not lead to severe clinical pathology despite a continuous inflammatory response in the CNS and the direct infection of neurons by the parasite. It therefore provides a useful model to address mechanisms of neuro-immune balance. The work here collectively characterizes a population of chronic neutrophils in the brain identified as “CBNeuts” during Toxoplasma infection that persists long after chronicity has been established. Depletion studies confirm the importance of neutrophils for the control of parasite replication in the brain and demonstrate that these neutrophils are significant sources of neuroprotective molecules and aid in neuronal repair. CBNeuts are phenotypically distinct from peripheral neutrophils and display CNS-specific heterogeneity at the transcriptional level indicating potential balancing of classical infection control and alternative neuroprotective functions during chronic Toxoplasma infection. While it is known that neutrophils are important during acute and early chronic infection, we now show that this is a highly persistent population in the brain and is therefore unlikely to be a first responder population either to parasite infection or initial invasion of the CNS. This population remains in the brain for the duration of chronic infection after a brief drop in both percentage and total cell numbers from 2- to 4wpi (Fig. 1). This drop is evidence of a lull between acute and chronic neutrophils and specifically points to the development of a long-lasting chronic neutrophil population after initial die-off of acute infiltrating cells during early chronic infection once parasites have formed chronic cysts inside neurons. Despite their low expression of the proliferative marker Ki67 (Supplemental Fig. 1B), the increase seen in neutrophil percentage and number from 4- to 6wpi suggests that these cells may be capable of either proliferation in the brain or are replenished from the circulation once chronic infection is established. This is also supported by increased numbers seen at 11wpi, when reactivation of the parasite can be seen prompting increased neutrophil infiltration to control parasite replication.

While there is evidence for a role of more “classical” antimicrobial functions by CBNeuts to directly control parasite replication during chronic infection, the data presented here introduce an alternative function of CBNeuts to produce NRG-1 and assist in neuronal repair mechanisms. NRG-1/ErbB4 signaling-dependent neuroprotection is vital for host survival in instances of CNS damage such as ischemic stroke, spinal cord injury, epilepsy, and experimental cerebral malaria infection [22, 23, 41–43]. Our study also demonstrates the ability of NRG-1/ErbB4 signaling to rescue infection-induced neuropathology in the form of GLT-1 loss via exogenous NRG-1 treatment during Toxoplasma infection, a neuroprotective mechanism that has not been reported previously. NRG-1 production has been broadly characterized predominantly in CNS-resident cells, specifically neurons and microglia [44–46]. However, expression of NRG-1 by migrating peripheral immune cells is less understood, and expression of NRG-1 and or NRG-1/ErbB4 signaling by neutrophils has not been previously documented. The recruitment of NRG-1-expressing immune cells may be an innate response to injury or an indication of the degree of CNS insult and therefore a need for additional peripheral support. CBNeuts were almost uniformly positive for NRG-1 protein although scRNAseq suggests that AE neutrophils are higher expressers of NRG-1 transcripts. It is important to note that neutrophils were not the only immunological source of NRG-1 as macrophages and CNS-resident microglia also expressed NRG-1 and ErbB4. In contrast, adaptive lymphocytes were rarely sources of these molecules. There is increasing recognition of the connection between the nervous and immune systems, however, this data points to a niche for adaptive T cell immunity being primarily about controlling infection and immunomodulation and not neuroprotection at least via these mechanisms. Innate neutrophil production of neuroprotective molecules may indicate a shift in the needs of the brain once adaptive lymphocyte responses are established. This is supported by data demonstrating neutrophils pivoting to protective functions in the CNS following spinal cord and optic nerve injury, ischemic stroke, and retinopathy [37, 38, 47–50]. In addition, expression of both NRG-1 and ErbB4 by CBNeuts (specifically CD15-) indicates the potential for these cells to conduct autocrine and paracrine signaling through the NRG-1/ErbB4 pathway. These results also suggest that this pathway could function as a feedback loop telling CBNeuts when to shift to a more neuroprotective vs. antimicrobial population.

In other systems, neutrophils have been defined as “alternative” and have functions that aid in angiogenesis and tissue repair as reviewed by Peisler and Kubes [15]. The presence of different neutrophil subsets in the brain during Toxoplasma infection supports the newly acquired appreciation for neutrophil heterogeneity which can be dependent on a multitude of factors including age and tissue environment [16–18, 30, 31]. Our work suggests that despite the common urge to classify immune cells as “N1” or “N2” in terms of function, these neutrophils are capable of acting as both anti-parasitic and tissue reparative in the brain microenvironment. There was no enrichment of effector genes *Mpo* or *Nox1* (encoding for NADP oxidase) in either AE or RR neutrophils, likely reflecting the significant post-translational regulation of these effector processes. However, CXCR4^hi^ AE neutrophils have upregulated transcription of the effector genes *Bmf* and *Pdgfb* and also *Vegfa*, and *Kdr*, noted for their roles in tissue repair. The overlap of these seemingly contradictory functions is steadily increasing with the recognition that the production of NETs and peroxidases are also required for tissue repair as well as effector mechanisms against pathogens [38, 47, 51, 52]. This underpins the concept of innate immune cells being as critical to tissue homeostasis as they are in initiating immune responses and may pinpoint neutrophil flexibility as important not only in Toxoplasma infection but also in all models of CNS disease.

Our work not only identifies CBNeuts as previously unknown sources of neuroprotective molecules but also demonstrates distinct subsets that are specific to the brain environment. Previous work has identified two broad categories of neutrophils that differ in their functions. The first is a “classical” pro-inflammatory subset that is associated with classical macrophage activation, increased granularity, NETosis, and the production of Th1 cytokines IL-12 and TNFα [13, 14]. The second class is considered “alternative” based on anti-inflammatory cytokine expression like IL-10 and IL-4, association with alternative macrophage activation, decreased granularity, and angiogenesis and vascular repair [12, 13]. Here, we demonstrate the presence of two major CBNeut subsets that differ from peripheral neutrophils suggesting that tissue-specific signals may drive the differentiation or retention of neutrophil subsets at the site of infection. These CBNeuts can be differentiated into two main populations, CXCR4^hi^ AE cells and recently recruited (RR) CXCR4^lo^ cells that have increased enrichment for *Fut4* indicating their increased migration potential [15]. It should be noted that corresponding *CXCR4*-enriched AE cells show decreased Ly6G expression in heatmap comparisons of gene expression between AE and RR populations in Fig. 3B. This difference in *Ly6G* enrichment between these subsets stems from the use of RR cells as the denominator group for these direct comparisons between populations. As the RR population has a higher *Ly6G* enrichment, this results in the low expression seen in the AE group. This is supported by previous work demonstrating that as neutrophils age, their expression of Ly6G decreases in preparation for recycling back to the bone marrow [15, 53]. Although both AE and RR CBNeuts exhibit signs of functional heterogeneity, the latter RR population has an enhanced effector signature indicated by increased expression of genes and GO terms associated with canonical pro-inflammatory and anti-microbial functions including “chemokine production” and “neutrophil-mediated killing of gram-negative bacterium.” This supports the hypothesis that as neutrophils enter the brain during infection, they are primarily there as an anti-microbial responsive unit. However, as infection becomes established with reduced parasite replication and cell lysis, neutrophils age and tissue-specific repair signals dominate.

The evidence for heterogenous functions of neutrophils in disease stems partially from their discovered roles in neuronal and angiogenic repair in both spinal cord and optic nerve injury demonstrating direct and indirect protection [37, 38, 48, 51, 54]. These functions discovered in non-infection instances may be applicable to the demonstrated evidence of damaged neurons observed during chronic Toxoplasma infection [19, 20, 55]. Additionally, the continuous latent reactivation of parasitic cysts in the brain may not only require the continuous presence of these protective neutrophils but may also be a stimulus for them to remain in the brain and differentiate into reparative subsets. As such, their consistent presence throughout chronic infection may ultimately be a signal of ongoing neuronal damage which is supported by slowly worsening neuronal function and changes in behavior over time [19, 56] and suggests increased plasticity of neutrophils in the brain and the dependence of neutrophil function on brain microenvironment. The infection-induced changes in neuronal circuitry indicate an increased need for neuronal repair attempts as well as increased infection control as chronicity progresses. The recruitment of innate immune cells such as neutrophils may be necessary for this process as specific neuronal circuitry is required to mobilize neutrophil migration from the bone marrow [57, 58]. Previous work has demonstrated the recruitment of innate inflammatory monocytes to the olfactory bulb of the brain perhaps to remedy olfactory-dependent behavioral changes [11]. Neutrophil localization to this area has been observed during homeostasis in zebrafish models [28] but was not observed in our murine model of Toxoplasma infection. While our work demonstrated no evidence of neutrophil localization in the brain, other changes in neuronal function may be dependent on neutrophil repair of previously damaged and re-wired circuits.

The communication between the brain and periphery that continues to draw in non-aged neutrophils (RR) even after 11wpi will be important to understand and may require parasite recrudescence and continued CCL2-dependent recruitment [59, 60]. In addition, a small cluster of aged neutrophils is observed in the spleen. This would either indicate differentiation of these cells in the periphery prior to migration to tissues or trafficking of tissue-educated neutrophils back into circulation and secondary lymphoid compartments. Such a population could act as an indication of continued CNS inflammation and thereby be a stimulus for new neutrophil recruitment. Whichever way these neutrophils differentiate, the question still remains as to what signals drive the brain tissue specificity of these cells and what factors encourage the differentiation into a protective phenotype. It is possible that neuronal antigens are somehow travelling to peripheral sites to initiate this process, but the mechanisms behind this subset differentiation remain to be elucidated.

Collectively, this study demonstrates a persistent neutrophil population in the brain throughout the duration of chronic Toxoplasma infection termed “chronic brain neutrophils” (CBNeuts). The depletion of these cells during chronic infection leads to enhanced infection and infection-induced neuropathology indicating a previously unknown role of neutrophils in maintenance of brain homeostasis during infection. Expression of NRG-1 by CBNeuts reveals a distinct brain-specific phenotypic and transcriptomic profile of these cells which also display functional heterogeneity based on age and integrin expression, and NRG-1/ErbB4 signaling play a role in limiting infection-induced neuropathology. In conclusion, these chronic brain neutrophils may represent a vital flexible component of the balanced immune response to chronic infection in the brain that *Toxoplasma gondii* is only one example of. Such capacity for functional flexibility within these cells marks them as potential therapeutic targets for enhanced neuroprotection during chronic Toxoplasma infection and presents a paradigm shift in our broader understanding of neutrophil functions in the brain as a whole.

## Electronic supplementary material

Below is the link to the electronic supplementary material.

Supplementary Material 1

## Data Availability

The data presented in this study are deposited in the Gene Expression Omnibus (GEO) repository (link: https://www.ncbi.nlm.nih.gov/geo/) and are accessible through GEO Series accession number GSE210883 (https://www.ncbi.nlm.nih.gov/geo/query/acc.cgi?acc=GSE210883).
