# Supplementary material for: Toxoplasma infection induces an aged neutrophil population in the CNS that is associated with neuronal protection
Source: J Neuroinflammation. 2024 Aug 2;21:189. doi: 10.1186/s12974-024-03176-7 (PMC11297776; doi:10.1186/s12974-024-03176-7)
Supplement: Supplementary file 1 — Supplementary Material 1 [file 12974_2024_3176_MOESM1_ESM.docx]

**
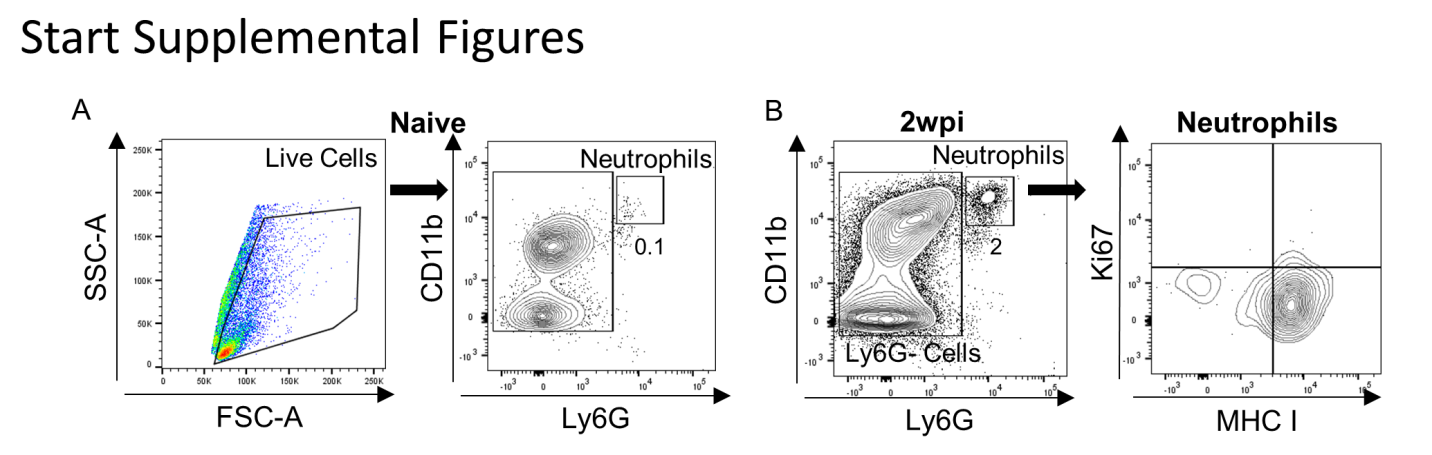
**

**Supplemental Figure 1. Neutrophil identification and gating strategy.** A) Representative gating strategy and gating of CD11b+Ly6G+ cells from the brain of naïve controls. Neutrophils were defined as CD11b+Ly6G+ after gating on live cells. Numerical values represent average percentages. B) Representative gating of CD11b+Ly6G+ cells from the brain of Toxoplasma-infected mice at the early chronic stage of infection (2wpi). Mature, non-proliferating neutrophils were then defined as Ki67-MHC I+. This gating strategy was also used for identification of neutrophils from peripheral spleen and blood.


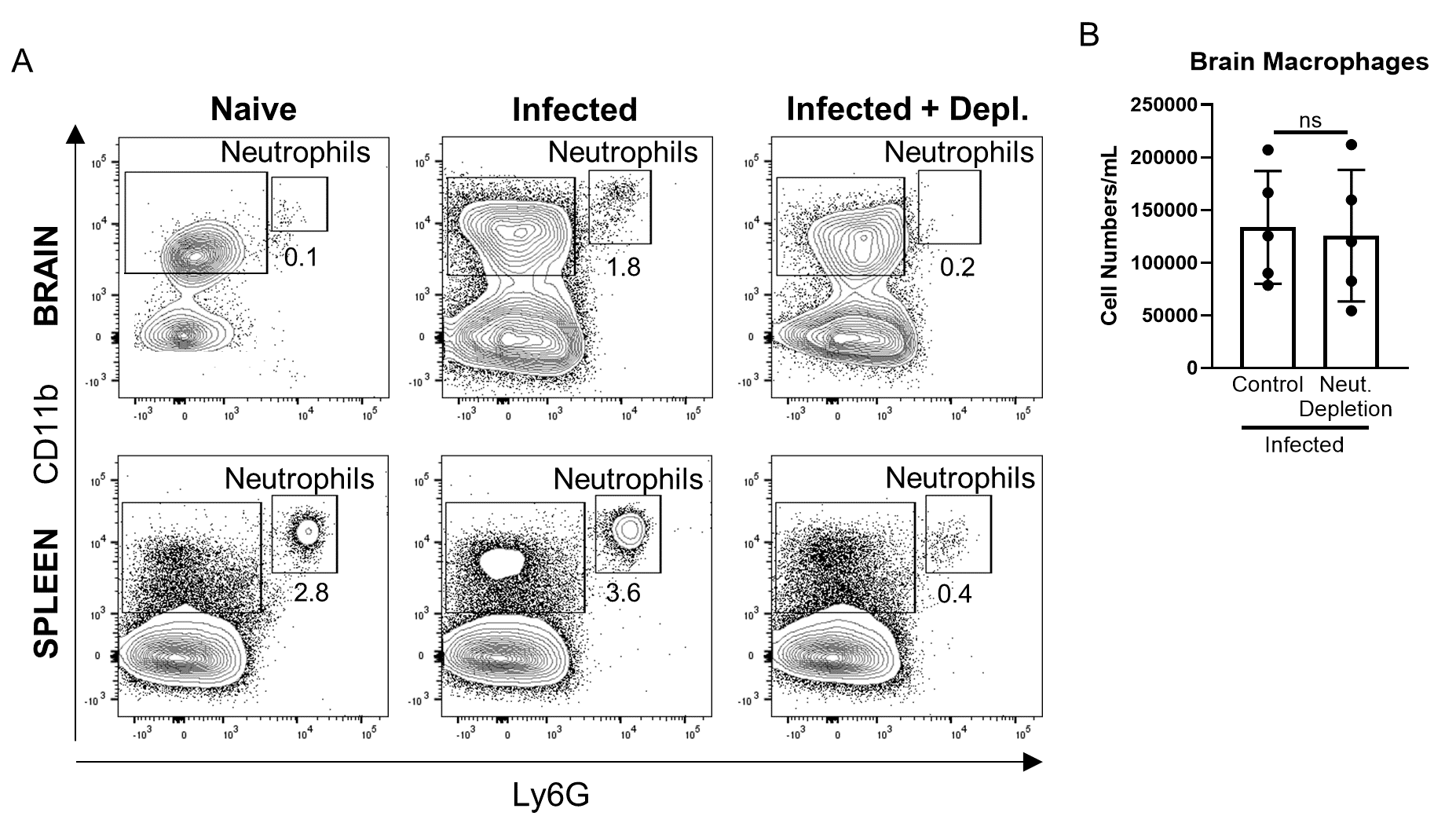


**Supplemental Figure 2. Successful and specific depletion of neutrophils after treatment with neutralizing Ly6G monoclonal antibody.** A) Representative gating of CD11b+Ly6G+ neutrophils from the brain and spleen of naïve controls, infected controls, and infected neutrophil-depleted mice. Neutrophils were defined as CD11b+Ly6G+ after gating on live cells. Numerical values represent average percentages. B) Number of macrophages in brain following Ly6G depletion treatment demonstrates neutrophil-specific depletion.


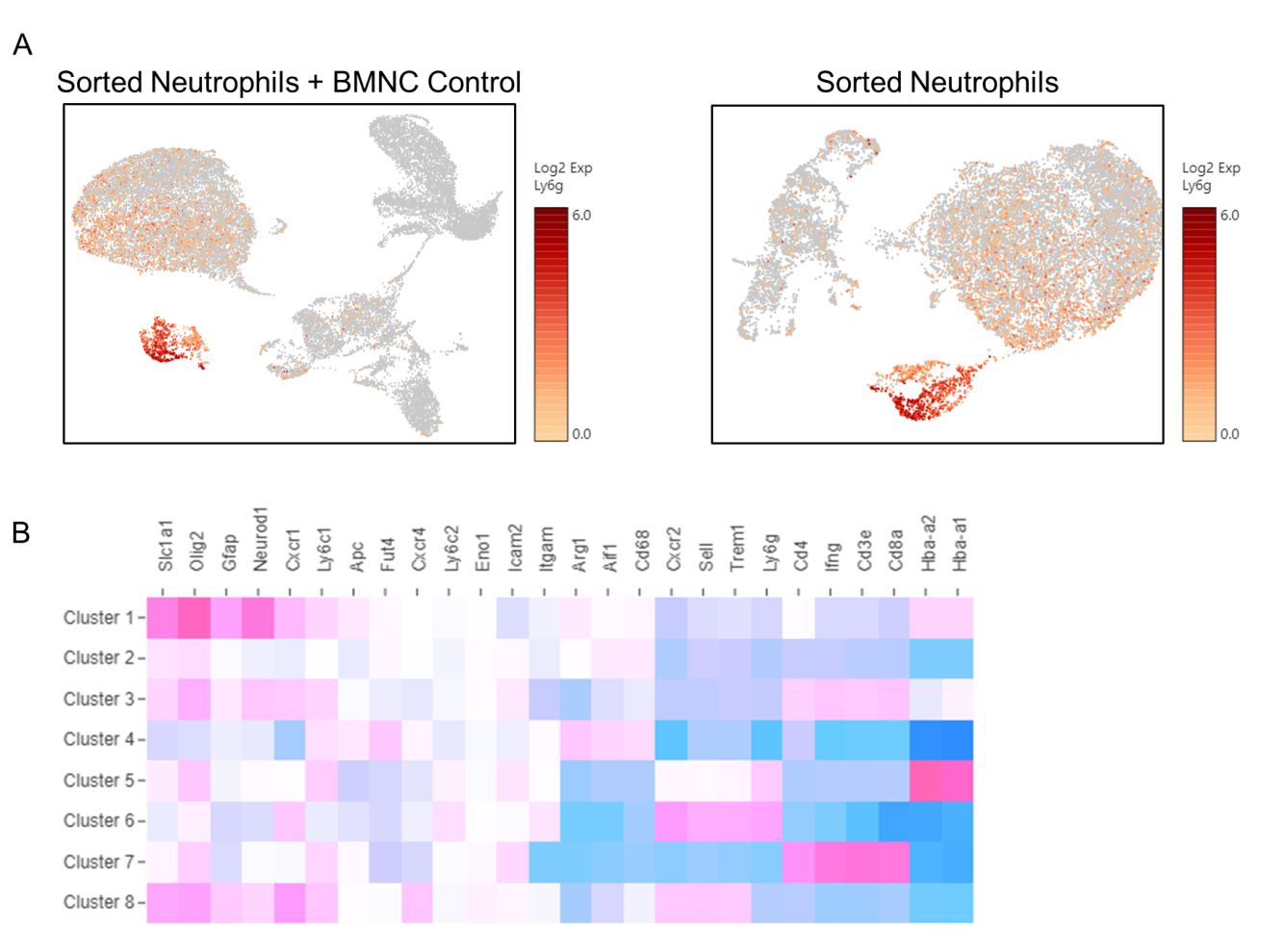


**Supplemental Figure 3. Identification of neutrophil and non-neutrophil signatures from scRNAseq data.** A) Ly6G log_2_ gene expression of respective aggregated UMAPs corresponding to Figure 2 to confirm positive neutrophil phenotype. B) Heat map of canonical markers of various cell types found within the sorted “CBNeut” population identified via scRNAseq analysis. Visualization of gene expression identified several cell types: red blood cells (Cluster 5, pink subset in Figure 3A), resident CNS cells (Cluster 1 and 8), T cells (Cluster 7), and unknown/potential apoptotic cells (Cluster 2 and 3) respectively.

**Supplementary Table 1. Flow cytometry antibody panels.**

| **Name of Panel** | **Primary Antibody** | **Secondary Antibody (if applicable)** |
| --- | --- | --- |
| **NRG-1 and ErbB4 Kinetics (BD FACS Canto II)** | ErbB4 OR CD3 FITC | N/A |
|  | CD45 PE | N/A |
|  | Ly6G (Clone 1A8) PerCPCy5.5 | N/A |
|  | RbαM NRG-1 (SMDF) | DkαRb Alexafluor 647 |
|  | CD15 OR Ki67 Biotin OR CD8 PECy7 | Streptavidin PECy7 OR N/A |
|  | CD11b OR CD4 APCCy7 | N/A |
|  |  |  |
| **MSR1 Kinetics (BD FACS Canto II)** | RbαM MSR1 OR CD3 FITC | DkαRb Alexafluor 488 |
|  | CD45 PE | N/A |
|  | Ly6G (Clone 1A8) PerCPCy5.5 | N/A |
|  | CD11b APC OR RbαM MSR1 | N/A OR DkαRb Alexafluor 647 |
|  | CD15 Biotin | Streptavidin PECy7 |
|  | CD62-L OR CD4 APCCy7 | N/A |
|  |  |  |
| **Neutrophil Depletion (BD FACS Canto II)** | CD3 FITC | N/A |
|  | CD45 PE | N/A |
|  | Ly6G PerCPCy5.5 | N/A |
|  | CD4 APC | N/A |
|  | CD8 PECy7 | N/A |
|  | CD11b APCCy7 | N/A |
|  |  |  |
| **Alternative Protein Phenotyping (Novocyte)** | ErbB4 FITC | N/A |
|  | MαM NRG1 (SMDF) | DkαRb Alexafluor 568 |
|  | CD11b PerCPCy5.5 | N/A |
|  | MMP9 Alexafluor 647 | N/A |
|  | CD15 Biotin | Streptavidin PECy7 |
|  | CD62-L APCCy7 | N/A |
|  | Ly6G (Clone 1A8) BV510 | N/A |
|  | RbαM MSR1 | DkαRb Qdot655 |
|  | GtαM VEGF | DkαGt Alexafluor 680 |
|  | CXCR4 PECy5.5 | N/A |

**Supplementary Table 2. Immunofluorescence antibody panels.**

| **Name of Panel** | **Primary Antibody** | **Secondary Antibody (if applicable)** |
| --- | --- | --- |
| **Brain SLPI Panel** | RbαM SLPI | DkαRb Alexafluor 488 |
|  | GtαToxoplasma | DkαGt Alexafluor 568 |
|  |  |  |
| **NRG-1 Treatment Panel** | GpαM GLT-1 | ChkαGp Alexafluor 488 |
|  | GtαToxoplasma | DkαGt Alexafluor 568 |
